# Influence of Internet and Social Media in the Promotion of Alternative Oncology, Cancer Quackery, and the Predatory Publishing Phenomenon

**DOI:** 10.7759/cureus.2617

**Published:** 2018-05-13

**Authors:** Pedro David Delgado-López, Eva María Corrales-García

**Affiliations:** 1 Neurosurgery, Hospital Universitario De Burgos, Burgos, ESP; 2 Radiation Oncology, Hospital Universitario De Burgos, Burgos, ESP

**Keywords:** quackery, predatory journals, social media, internet, complementary and alternative medicine, oncology

## Abstract

In the last decade, electronic media has irrupted physician’s clinical practice. Patients increasingly use Internet and social media to obtain enormous amounts of unsupervised data about cancer. Blogs, social networking sites, online support groups and forums are useful channels for medical education and experience sharing but also perfect environments for misinformation, quackery, violation of privacy and lack of professionalism. The widespread availability of such electronic resources allows some followers of the alternative oncology to spread useless irrational and controversial remedies for cancer, like false medicaments, miraculous diets, electronic devices, and even psychic therapies, as did charlatans in the past, providing false expectations about cancer treatments. Moreover, so-called predatory journals have introduced confusion and malpractice within the academic biomedical publishing system. This is a rising editorial phenomenon affecting all fields of biomedicine, including oncology that jeopardizes the quality of scientific contribution and damages the image of open access publication.

## Introduction and background

Patients are increasingly using the Internet and social media to obtain medical information, especially about cancer treatments [1-3]. These novel methods for science divulgation lack specific regulatory norms and are sheltered by anonymity and absence of professional supervision. Blogs, social networking sites and other electronic resources are valuable methods for oncologic education but also perfect environments for misinformation and quackery. Additionally, there is a flourishing and often fraudulent business regarding alternative oncologic therapy given the hopelessness and psychological vulnerability that many cancer patients suffer at some point of their disease [4].

In the era of electronic media, the influence of leading doctors is paramount [5]. The promotion of healthy habits and proven therapies by reputed medical institutions is often confronted with the irrational and sometimes dangerous advice provided by irresponsible caregivers, not to mention the pernicious influence of uninformed or ignorant celebrities [6].

The concepts of Complementary and Alternative Medicine (CAM) and Integrative Medicine are increasingly gaining relevance among caregivers, particularly in the oncology field [4,7-9]. These are interesting clinical phenomena with a double dimension: they intend to improve or minimize the adverse effects provoked by conventional anti-cancer therapies, but may also promote implausible effects, often linked to dishonest financial profits.

Additionally, a new and rising editorial phenomenon called predatory publishing is threatening the quality of scientific contribution and undermining the image of open-access publication [10]. Predatory journals lack appropriate editorial methodology, essentially regarding peer reviewing, and promote the spreading of pseudoscience, conspiracy theories, low-quality studies, plagiarism, false data, and introduce confusion and malpractice in biomedical research [11,12].

Oncology is not immune to online misinformation, quackery and predatory publishing. Many examples of unscrupulous and unethical behavior can be found in the past and at present [6,13]. In this paper, we review the characteristics and influence of the Internet-based resources and social media in cancer treatment, describe the concepts and repercussion of alternative oncology and cancer quackery, and highlight the importance of recognizing predatory publishing in our current oncologic practice.

## Review

Pros and cons of online oncologic consultation

Medical appointment, the usual channel of communication between physician and patient, has been progressively complemented, and sometimes substituted, by novel methods of obtaining medical advice supported by the widespread availability of Internet-based electronic resources [14-16]. These powerful tools provide all kinds of medical and oncologic data and help to spread specific therapy recommendations among users. Unlike classical professional medical consultation, electronic communication is by definition an unsupervised source of information that lacks provable quality control.

The growing volume of clinically relevant oncologic data, currently published in more than 200 oncologic journals, makes it infeasible for any single physician to remain updated [1,3]. Additionally, there seems to be a transition from anatomically defined heterogeneous cancer patient groups, like breast cancer or lung cancer, to molecularly defined cancer subgroups, with specific and unique characteristics [1]. Patients use the Internet for seeking information about their particular cancer subgroup and for connecting to other patients and caregivers looking for emotional support, mainly through social media. In general, the confidence of the lay public on healthcare professionals is still high in comparison to that obtained from online resources, although the Internet seems to be the preferred method for obtaining emotional support and also for sharing practical advice with other cancer patients [1].

In social media, users are the actual creators of content, who share information in a virtual community environment [1-3]. Thus, concerns about professionalism and patient privacy have arisen among healthcare providers regarding the use of social media [2,3]. Nevertheless, online resources offer new opportunities for massive dissemination of valuable and validated medical information, via online groups, blogs, forums, and conversational platforms like social media. As an example, educational video clips about chemotherapy treatment and balanced dietary habits posted on a YouTube channel, along with a supportive medical blog, have shown efficacy in spreading cancer-related issues [17].

Self-aggregating online groups of cancer patients have emerged as part of the clinical decision-making scheme and recent surveys suggest that the beneficial effects of online consultation seem to outweigh their potential harmful effects [1]. Electronic consulting has facilitated faster enrollment in clinical trials and favored patients to travel to advanced oncologic treatment facilities [18]. Additionally, patients are now able to pool their own data (especially so-called patient-reported outcomes or PROs, considered extremely valuable endpoints in clinical trials) in international or multicenter aggregated databases that may help to corroborate clinical observations or generate new hypothesis for future trials [19].

The American College of Physicians and the Federation of State Medical Boards have published recommendations about the influence of social media on the patient-physician relationship, the role of these media in public perception of physician behaviors, and strategies for doctor-to-doctor communication that preserve confidentiality [20]. A recent systematic review by Maher et al. [21] concluded that there is still very modest evidence that health behavior change interventions incorporating online social networks may be effective, largely because the effect sizes were small in magnitude and statistically non-significant.

However, unsupervised online oncologic advice is a potential threat to cancer patients for several reasons [1,3]. First, wrong advice or misinformation may hinder cure or worsen prognosis in certain cancer patients. Second, electronic platforms may serve as a background for parallel profitable business activity aimed to sell useless remedies or recommend expensive unproven therapies. Third, the Internet provides an unorganized mixture of non-contextualized oncologic data from a myriad of studies and non-professional opinions, which might be a source of false hope or psychological stress for patients and families. Fourth, it deteriorates the image of professional and recognized oncologists because the general public often cannot distinguish between reputed professionals and charlatans. This is partly due to the inherent complete lack of regulation or quality certification for websites or blogs. And finally, uncertainty about the origin and quality of information provokes a sense of general distrust in science.

Today, conventional oncologic therapies coexist with a series of other complementary, heterodox or implausible approaches to cancer, some of which are listed and briefly defined in Table 1.

**Table 1:** Glossary of definitions regarding unconventional therapies and approaches to cancer treatment.

Term	Definition
Conventional or standard oncologic therapy	Current validated cancer therapies include surgery, chemotherapy, radiotherapy, immunotherapy and combinations of them.
Complementary and alternative medicine (Oncology)	Refers to validated treatments aiming to help patients cope with the adverse effects provoked by conventional anti-cancer therapies (Complementary) plus other non-validated treatments that substitute conventional therapy with controversial or inexistent efficacy (Alternative).
Integrative medicine/oncology	The sum of conventional and complementary anti-cancer treatments. A holistic approach to cancer patients.
Unconventional therapies	All other therapies not included in the conventional or standard therapy.
Holistic therapies	Therapies that try to unify and combine the best of both conventional and complementary treatments. Addresses spiritual, psychological and physical needs.
Natural remedies	Non-pharmacologic therapies generally using medicinal herbs to prevent or treat disease or promote health.
Alternative medicine	Diverse health care practices, like homeopathy, whose tenets often differ from those of conventional medicine and are not generally taught in conventional medical schools. Scientifically unproven therapies.
Pseudoscience	Statement, belief or practice incorrectly presented as scientific and not supported by a valid scientific method, which cannot be reliably confirmed.
Oncologic charlatanism	Fake pretension of self-attributed medical or oncologic knowledge or skills.
Cancer quackery	The promotion of unproven or disproved cancer treatments instead of conventional therapies, usually within the context of a profitable business.
Conspiracy oncology	Irrational belief that drug companies and government agencies suppress alternative cures for cancer in their own benefit.

Internet-based resources and social media in oncology

The first electronic method for patient-doctor communication was the email. It served as a much faster surrogate for conventional mail, assuming however a significant gap between message’s arrival and the eventual action taken by the doctor [3]. Partly because it is free of charge, email has been overused and a relevant proportion of the messages received is actually neglected. Although reasonably safe, complete privacy of email cannot be fully guaranteed. Listservs (mailing lists) were used to disseminate information via email to many users at a time, in order to make cancer patients feel part of a community and decrease their sense of isolation [3]. However, messages are sent only to individuals signing up for this service, and always to all of them. Individual users cannot selectively dismiss messages lacking interest unless they choose to be definitively removed from the mailing list. Although passive, listservs (an application that distributes messages to subscribers on an electronic mailing list) were in fact the precursors of current social networks.

*Blogs* (short for weblogs) allow people to share experiences, knowledge or feelings by writing a series of posts, usually listed in a reverse chronological order. Readers need to visit the author’s website to get the content. This passive and unidirectional method is meant to provide advice rather than promote conversation. Current platforms allow anyone to be a blogger regardless of academic or professional skills. Worldwide, the number of blogs exceeds several millions and those involving oncology are virtually countless. Again, the content of a blog is unsupervised and readers just follow what the blogger has to say.

However, blogs are reported to be helpful for cancer patients whether they are mere readers or the actual authors. An illness blog can be a vehicle of vital expression for people with advanced cancer [22]. Studies show that young adults and adolescents with cancer, accustomed to use the Internet and social media as a daily part of their lives, are empowered by the writing of blogs which seem to play a role in increasing well-being and a sense of coherence when confronting the existential issue of death [23]. Relatives of cancer patients also benefit from blogging. They are useful to find people with similar experiences, and help them in their grief process and to preserve memories. Contrarily, blogs can also be perceived by readers as tasteless “reality shows” [24].

The interesting study by Quinn et al. [25] about the influence of social media in cancer showed that breast cancer-related topics had significantly more posts per day compared to other cancers, the majority displayed on social media sites. However, information posted on blogs and discussion forums, especially anonymous posts, showed significantly lower levels of scientific accuracy compared to other electronic resources. According to a Swedish study [26], the healthcare personnel can also be subjectively influenced by the reading of a patient’s critical blog. In this study, the authors showed that staff exhibiting a neutral, professional and pragmatic attitude is more inclined to heed and respond to critic patients [26].

A controversial issue is whether blogs should be cited in scientific papers. Peer-reviewers are usually encouraged to check blog posts cited in manuscripts for relevance and credibility. The fact that authors can rapidly delete a post or an entire blog site, raises concern about the longevity of blogs compared to other forms of literature and brings into question the validity of the citation for future readers [27].

*Twitter* is a social media platform that allows unregistered users to read small messages up to 140 characters. Registered users can also post comments in a conversational-like fashion. It can be seen as an interaction microblog in which messages or other linked contents can be spread or retweetted, engaging in an online dialogue that can be massively amplified. The authenticity of an author’s tweet (@) is theoretically guaranteed by the system, and hashtags (#) serve as keywords for users’ search. The paucity of information contained in 140 characters has raised concern about its quality [3]. Oncologists also use Twitter to converse with one another, especially at professional meetings. However, concerns about privacy do exist: a percentage of tweets may contain private information of patients, or spread unprofessional comments about patients or colleagues [28]. Followers are not necessarily followed back in reciprocity so, as in blogs, information usually travels asymmetrically. Facebook allows registered users to share posts to previously authorized friends or to anyone including unregistered users.

Many examples on the use of Twitter in cancer patients can be found in the literature. The study by Sugawa et al. [29] has shown that so-called *Twitter powered accounts* (those with at least 500 followers) of cancer patients exhibited a majority of tweets focused on greetings, treatment discussion and psychological support, but surprisingly not centered on the dissemination of medical information. The study by Attai et al. [30], aiming to determine the effectiveness of Twitter as a tool for breast cancer patient education, demonstrated that breast cancer patients’ perceived knowledge increased and anxiety decreased by participating in a Twitter support group. Contrarily, a twitter-based campaign launched in Canada in 2013 aiming to raise awareness and understanding about prostate and testicular cancer failed to achieve significant conversations about men’s health and, specifically, about prostate and testicular cancers among patients [31].

The study by Owen et al. [32], among cancer survivors with psychological distress participating in an Internet-based intervention, showed that patients spent more time using social networks compared to the time spent following the structured intervention content, and greater engagement with the program was associated with previous chemotherapy and being female [32]. The authors concluded that social networks clearly improve engagement and they should be considered key platforms for oncologic Internet-based interventions.

Today, *Google* is doubtlessly the initial search strategy that patients use to obtain oncologic information [1]. A Google search returns thousands if not millions of entries for every particular cancer subtype. Users are literally overwhelmed with unvalidated, heterogeneous, non-contextualized and sometimes not scientifically structured information. Oncology has reached such a point of deep sub-specialization, that real expertise in interpreting the clinical significance of new advancements and cancer therapies is mandatory [1]. However, scientific articles found in Google searches are subject to quality and accessibility bias. Interestingly, older patients and people without an Internet connection are commonly alienated from this resource and kept away from what has been termed as *Dr Google phenomenon* [33].

In an effort to establish guidelines on the potential risks of using Internet-based resources as oncologic advice providers, Dizon et al. [34] published some recommendations regarding policies that may minimize the medico-legal issues related to social media interaction by encouraging the use of disclosures like: *social media content is not medical advice, responses may not be timely, accuracy of information is not assured, and communications are not confidential*. Although powerful in spreading information, current social media lack enough reliability and robustness as to provide professional, timely and secure medical advice.

Today, medical and oncologic advice from many times self-proclaimed *influencers* is a growing health and social issue with potential benefits and pitfalls. The great majority of influencers are not healthcare professionals nor have medical training or skills. Their knowledge comes from individual experiences or beliefs many of which are useless, irrational or unproven. They take advantage of the powerful tools of electronic communication to spread advice, often self-centered, and sometimes incurring in flagrant conflict of interest. Doctors can also be influencers on the basis of their expertise. Some of them claim to belong to some holistic or integral oncologic movement aiming to help patients cope with their suffering and existential stress [7-9].

Integrative oncology versus cancer quackery

*Integrative oncology* [8] is considered a respectable oncologic approach that complements conventional therapy, conceptually opposed to *Alternative oncology*. Integrative oncology addresses symptom control with non-pharmacologic therapies [8]. These complementary treatments are non-invasive but evidence-based adjuncts to conventional therapies that enhance control of physical and emotional symptoms. Contrarily, alternative oncology offers treatments without proven anti-cancer efficacy or safety. Thus, there may be some confusion with the acronym CAM (*Complementary and Alternative Medicine*): complementary and integrative approaches have demonstrated benefit and safety, but alternative therapies have not. In fact, if they were truly effective, they would not be alternative, but part of the conventional cancer care. Complementary therapies improve quality of life and also promote patients to have an active role and responsibility in their own care, which, in turn, enhances the doctor-patient relationship and their general well-being [9]. Some examples of complementary cancer therapies are listed and briefly commented in Table 2.

**Table 2:** Complementary oncologic therapies and brief description. (Modified from Deng and Cassileth [7], and Atwood [4]).

Complementary cancer therapies	
Meditation, relaxation	Practice aimed to focus mind on a particular object, thought or activity in order to achieve a mentally clear and emotionally calm state. Used in oncology to reduce anxiety and pain.
Biofeedback	A process in which electronic monitoring of a normally automatic bodily function is used to train a patient to acquire voluntary control of that function.
Acupuncture	An ancient Chinese medical practice aimed to treat illness or provide local anesthesia by the insertion of needles at specified sites of the body.
Mindfulness	A therapeutic technique that promotes a mental state achieved by focusing one's awareness on the present moment, while calmly acknowledging and accepting one's feelings, thoughts, and bodily sensations.
Homeopathy	A pseudoscience that claims that a substance that causes the symptoms of a disease in healthy people would cure similar symptoms in sick people.
Prayer	A personal solemn request for help or expression of thanks addressed to God or another deity.
Massage, chiropractic	Manual manipulation of spine, joints and other soft tissues aimed to improve musculoskeletal disorders and other conditions, especially pain.
Movement therapies (Yoga, Tai Chi, etc.)	A broad range of Eastern and Western movement approaches used to promote physical, mental, emotional, and spiritual wellbeing.
Art and Music therapy	Form of creative therapy in which the therapist guides the patient in using visual art as a form of expression or communication. Listening or more active participation in music aimed to improve quality of life. Both used to resolve personal conflicts and cope with grief, anxiety and stress.
Controlled physical exercise	Supervised exercise aimed to improve general physical state in a cancer patient.
Nutritional counseling	Professional dietitian providing advice and support regarding dietary needs and identifying areas where diet change is needed.

*Cancer Quackery* is defined as the promotion of unproven or disproved cancer treatments instead of conventional therapy [6]. The term quack comes from the ancient Dutch word *quacksalver*, meaning the person who used to sell wares on the market shouting loudly and who fraudulently pretended to have certain knowledge or skills. These people were also known as charlatans or snake oil salesmen.

Some examples of cancer quackery are listed in Table 3. As systematized by Cassileth and Yarett [6], categories of cancer quackery include potentially dangerous exotic dietary supplements and herbal remedies, useless complex chemical compounds, venoms and other related toxins, biologic products like shark cartilage extracts, oxygen-enriched therapies, supposed manipulation of human energy fields, shaman’s activity like healing touch or esoteric tutoring, electrical devices delivering curative electromagnetic fields and currents, emotional stress and mind-body connections techniques (beyond meditation and biofeedback), and prayer as an alternative (not as an adjunct) to mainstream cancer therapy. Special dietary supplements with vitamins (like the famous Laetrile [35] or vitamin B17) and coffee enemas are common features among different miraculous diets and remedies for all types of cancer. In the past, well-known physicians promoted the use of some of these diets, which eventually were also followed by current influent celebrities [6,9]. The website www.quackwatch.org is an international network of people focused on quackery-related information which provides reliable scientific websites for cancer advice and also allows to inform on frauds or inappropriate oncologic behavior.

**Table 3:** Historic examples of unproven cancer therapies and cancer quackery with a brief description. (Modified from Cassileth and Yarett [6]). FDA: Food and Drug Administration

Unproven cancer therapies and cancer quackery	
Laetrile [35]	Fake anti-cancer medication, also known as vitamin B17 or Amygdalin, introduced in the 1920s and popularized in the 1980s.
Shark cartilage	Developed in the 1950s. Reported anti-angiogenic action in vitro but not in clinical studies. Controversial bioavailability when consumed orally.
Electrical devices	Aimed to diagnose and treat all types of cancer with electromagnetic fields and currents. Borrows pseudo-scientific language from physics. Bioresonance therapy targets cancer cells, which emit specific and pernicious electromagnetic oscillations. Other devices try to balance the body’s bio-energetic forces or transform the modulated frequency patterns of the body into bioresonance magnetic frequency patterns.
Oxygen devices	Hyper-oxygenation is supposed to destroy cancer cells. Many administration options: oral, intravenous, colonic delivery of hydrogen peroxide, ozone-treated blood infusion. Unproven therapy with potential serious adverse effects and reported fatalities.
Herbs, naturopathic supplements	Essiac tea: a multi-herbal remedy popularized by a nurse named Ren Caisse in the 1920s (Essiac is her last name spelled backwards) still in the market. Untrue anti-cancer assigned properties. Entelev: a chemical mixture of several acids and other compounds developed in 1936. It supposedly balances the vibrational energy of cancer cells, causing them to self-digest and be expelled from the body. FDA made it illegal to distribute in 1989.
Prayer	Harmless and helpful to some cancer patients when used in conjunction with conventional treatment. However, prayer (or intercessory prayer) alone does not seem to have any impact on cancer prognosis.
Energy therapies	Based on the supposed existence of energy fields around the body. Examples: so-called Therapeutic touch by spiritual healers, application of electromagnetic fields with special devices, practice of voodoo techniques, etc.
Mind-body techniques	Patients can enhance mind power to restore body illness. Emotional conflict shocks are responsible for cancer induction that can be reversed with special psychic techniques aimed to balance the mind-body connection.
Kelley/Gonzalez regimen	150 supplements of vitamins, minerals, and pancreatic enzymes (from pigs) plus daily coffee enemas and special diets.
Gerson therapy	Organic, plant-based diet, raw juices, coffee enemas and natural supplements.
Immune-augmentative therapy	Injection of blood serum proteins that restores immune defenses and controls all forms of cancer.
Antineoplastons	Chemical compounds found normally in blood and urine made up of amino acids and peptides.
Cranial osteopathy	Gentle and relaxant cranial manipulation.
Reflexology	Technique claiming healing of body parts represented on the sole of the foot. However, simple foot massage can be relaxing.
Other	Plants and animal parts, fermented soy drinks, special oils Alkaline water, coffee enemas, antioxidants, megavitamins therapy, bee and scorpion venoms

Although a positive attitude can be beneficial when facing cancer treatment-related adverse effects, there is no scientific evidence supporting a demonstrable link between a certain psychological predisposition and cancer prognosis. Media events and population campaigns highlighting the great importance of attitude in overcoming the disease can be distressful for some cancer patients, especially those in advanced stages. They may feel pressured to carry the responsibility of their own cure. Distressed patients unable to believe in the cure for their disease may feel a sense of guilt for not being able to cope with the situation [6].

Finally, *Cancer conspiracy* is the belief that effective alternative cures for cancer are systematically silenced or suppressed by powerful drug companies or government agencies for their own benefit. This is an interesting issue promoted from certain websites included in the broader concept of global conspiracy. A paradigmatic example is the paranoid belief that so-called *chemtrails* (cloud trails produced by airplane reactor engines) are methods of population control [36].

What can be done to improve patient and physician oncologic criterion and oppose charlatanism? Education is paramount. Both patients and physicians need to hold a skeptic and critic approach to miraculous oncologic advancements. Patients should receive honest information about the real effectiveness and applicability of new cancer therapies, and they must demand frank and realistic information from oncologists.

Oncologists need to be scientifically updated. They should not however incur in what we call the *last trial syndrome*: physicians feeling compelled to provide patients with the best possible treatment, assuming that the last trial protocol published is necessarily the best option. As medical history often recalls, *last is not always best*. The abuse of *off-label *oncologic medication can be an example of this tendency to grant the patient with any plausible therapy [37,38]. Doctors should avoid raising false hopes but also extreme pessimistic views, which is a perfect excuse for seeking advice from charlatans.

Patients are encouraged to visit only professional websites from reputed institutions or doctors, and to avoid personal and unprofessional blogs, especially if there are suspected individual or financial implications. Certain websites like https://www.nccn.org from the National Comprehensive Cancer Network or https://www.mskcc.org from the Memorial Sloan Kettering Cancer Center are valuable evidence-based sources of oncologic information suitable for patients.

Ethics committees and professional oncologic societies should be responsible for oncologic education and the promotion of healthy habits. They should lead academic persecution of charlatans and inform on illegal or immoral practices. Maybe professional oncologic societies should not only persecute misinformation but also focus on offering the same kind of information/communication patients are demanding and finding elsewhere, but doing so not only rigorously but also in an accessible way so to compete with the unofficial channels.

Predatory journals in oncology

Many oncologists receive lots of academic spam email with polite invitations for manuscript submission and professional meeting attendance [39]. As in the authors’ experience, these invitations can exceed 300 per month. Most of them are repetitive, often irrelevant and difficult to avoid or prevent [40]. This can be bothersome and confusing, especially for young oncologists unaware of the predatory publishing phenomenon.

Predatory publishing is a rising and profitable editorial business model based on the publication of scientific manuscripts in an open access fashion, in which the authors are who actually pay for the publication process. Predatory journals lack adequate editorial methodology: transparency, definition of editorial policy, peer-review process, established and recognizable editorial boards, realistically assigned impact factor, explicitly stated country of origin, inclusion in reliable and solid databases (like Scopus or PubMed), and usual financial features like reasonable subscription and open access publication fees [10-12,41]. Most predatory journals operate via websites located in many developing countries like India, Pakistan or Nigeria, but also from the United States or the United Kingdom. This phenomenon was described in 2010 by Jeffrey Beall, a librarian from Denver University in Colorado [11]. He collected a large amount of unwanted academic spam email and elaborated a list of potentially predatory journals and publishers. The number of journals appearing in that list (now replaced by the website www.predatoryjournals.com) continuously increases. Currently, legitimate biomedical publications are massively outnumbered by predatory journals. The preferred content of predatory journals is related to pharmacologic and engineering issues although every medical sub-specialty, including oncology is involved. Noticeably, predatory journals’ names are strikingly similar to legitimate ones, probably in an effort to increase credibility and prestige [41].

The study by Shamseer et al. [42] identified 13 evidence-based features by which predatory journals can be distinguished from legitimate journals (see Table 4). They are usually focused on general biomedical and non-biomedical research with a broad scope of interest in which virtually any contribution may fit. They promise very rapid editorial process, the manuscript handling is undefined or inexistent, fail to mention a copyright policy, the website contains many spelling and grammar errors, and the contact email address is usually non-professional (e.g., @gmail.com or @yahoo.com). Predatory journals promote the Index Copernicus value instead of the Journal Citation Report Impact Factor, the article publication charge is surprisingly low (commonly < 150 US$) compared to open access legitimate journals (>1500-2000 US$), and there is no retraction policy.

**Table 4:** Main features distinguishing a typical predatory journal from a legitimate journal. (Modified from Shamseer et al. [42]).

Features	Comments
Scope and appearance	Broad-range scope with general themes including non-biomedical subjects. Journal names are very similar to legitimate but with slight differences. Website contains spelling and grammar errors. Unprofessional appearance. Contact address is non-professional or non-journal affiliated (e.g., @gmail.com or @yahoo.com). Images are distorted, unauthorized, or intended to look something they are not.
Editorial policy	There is no defined editorial policy. No retraction policy. No information about digital content preservation. Homepage language targets authors instead of potential readers. Rapid publication (within days) is always promised.
Country of origin	Not always clearly defined. Most websites from developing countries.
Editorial committee	Inexistent or fraudulent. Reputed editors may inadvertently belong to these committees.
Financial support	Authors are charged for the processing/publication process. Fees for open access publication are relatively low (usually <150 US$).
Manuscript handling	The manuscript handling process is not clearly described. Peer review process does not exist or it is obscure.
Open access	Always offer Internet open access publication.
Impact factor	Index Copernicus Value is promoted: a controversial index created in Poland in 1999 as an alternative to English-language indexes like the classical Journal Citation Report Impact Factor from Thomson Reuters.
Copyright policy	Open access publications either retain copyright of published research or fail to mention a specific copyright policy.

The combined search of MeSH terms “predatory” and “oncology” in a PubMed search, including related articles, yields virtually no information except for very few papers on the matter [42-44]. Most of the spam emails received by oncologists are invitations to international oncology meetings and requests for manuscript submission relating any aspect of all sub-specialties, both in clinical and radiation oncology. A close look at the names of predatory journals in Beall’s list shows that a relevant percentage of them refers to general oncologic aspects, chemotherapy, pharmacology and radiation physics [43]. The recently published study by Clemons et al. [44] is paradigmatic of how physicians and academic researchers are increasingly harassed with spam invitations. The majority of spam emails received by an average academic medical oncologist over a three-month period (N = 156) included invitations to submit to predatory journals, invitations to predatory conferences, advertisements, and surveys.

This phenomenon has reached an unbelievable and absurd dimension. In 2005, David Mazières and Eddie Kohler, two American researchers tired of receiving unwanted spam, wrote a paper titled *Get me off Your F--king Mailing List* and submitted it to an international informatics conference [45]. The paper just consisted of that sentence repeated hundreds of times but structured as a research article! Amazingly, in 2014, the International Journal of Advanced Computer Technology peer-reviewed that manuscript and rated it as excellent, and accepted it for publication under a US$150 charge [46].

What can be done against predatory publishing? First, improve so-called *publishing literacy*, meaning to increase the knowledge on biomedical research methodology including biostatistics, ethics, and other scientific issues, especially among younger authors [47]. Second, do not respond to suspicious emails and proposals for collaboration away from renowned editorial environments [48]. Third, it is important that academic institutions provide with consensual statements about the curricular credit of legitimate and predatory publications and reduce the pressure for publication on young researchers in order to obtain academic grants or postgraduate research projects. Younger authors from developing countries are at higher risk of incurring in predatory authorship [49]. An accreditation system similar to that required for any industry producing goods (scientific/medical knowledge actually is), with periodic audits might be required (or at least available) for scientific/medical journals.

On the other hand, reputed medical and oncologic institutions, including national and regional specialty societies, leading journals publishers, and ethics committees in hospitals should ideally provide authors with detailed information about the predatory publishing phenomenon, how to recognize it and avoid it. Initiatives like *http://thinkchecksubmit.org/ *help authors to choose the appropriate journal for each manuscript [48]. Likewise, consolidated databases like Scopus, PubMed and Web Of Science, should ideally eliminate fraudulent journals from their electronic indexation resources. Currently, many alleged predatory journals manage to appear in databases like Google Scholar and even in PubMed.

## Conclusions

Electronic patient to doctor communication is currently evolving. The Internet seems to be a valuable platform for oncologic education although its use implies potential threats. The power of electronic resources for massive dissemination of useful oncologic contents is often confronted with the potential spreading of unwanted, unsupervised and sometimes harmful information. Currently, blogs and social networks are preferentially used for exchanging experiences and emotional support rather than diffusion of true scientific oncologic information.

Since Internet users are the actual creators of content, scientifically unsupervised data can be a source of potential harm for cancer patients. Complementary oncologic therapy is considered an evidence-based and helpful adjunct aimed to improve symptoms attributable to conventional cancer treatments. However, alternative oncology tries to substitute proven oncologic therapy and can be detrimental and dangerous.

Cancer charlatanism is a well-documented fraud that can be reversed with medical education. Both doctors and patients should hold a skeptic and critic approach regarding miraculous oncologic advances. Oncology and general medical institutions should play a leading role in opposing cancer quackery. The predatory publishing phenomenon in oncology needs to be recognized and opposed. It threatens the quality of scientific contribution, and damages the image of legitimate and high-quality open access oncologic journals.
